# The Differential Impact of Screen Time on Children’s Wellbeing

**DOI:** 10.3390/ijerph18179143

**Published:** 2021-08-30

**Authors:** Sarahjane Belton, Johann Issartel, Stephen Behan, Hannah Goss, Cameron Peers

**Affiliations:** 1 School of Health and Human Performance, Dublin City University, D09 NA55 Dublin, Ireland; johann.issartel@dcu.ie (J.I.); stephen.behan@dcu.ie (S.B.); hannah.goss@dcu.ie (H.G.); 2 INSIGHT Centre for Data Analytics, Dublin City University, D09 NA55 Dublin, Ireland; 3 Faculty of Science and Health, Dublin City University, D09 NA55 Dublin, Ireland; cameron.peers@dcu.ie

**Keywords:** leisure screen time, quality of life, health, wellbeing dimension, BMI, youth

## Abstract

Increased screen time has been found to be associated with a number of negative health and wellbeing indicators in youth populations. An increasing number of studies have investigated the association between screen time and wellbeing in adolescents, but evidence in younger children is still emerging. This 2017 study explored the effect of leisure screen time and gender on dimensions of wellbeing (measured using KIDSCREEN-27) in a national sample of 897 Irish primary school children aged 8–12 years. Participants had a mean age of 10.9 ± 1.16 years and were 47.7% female. Just over 30% of the sample accumulated 2 h or more of leisure screen time daily. Results show that there was no significant interaction between screen time category (<2 h/2 h + daily) and gender on overall wellbeing, while controlling for BMI. Children who self-reported <2 h of leisure screen time scored significantly higher on four dimensions of wellbeing: physical, parental, peers, and school, but not psychological. This study supports the growing evidence of the impact that leisure screen time has on health. Further longitudinal research investigating the impact of sub-categories of leisure screen time behaviour on wellbeing is warranted.

## 1. Introduction

Screen time specifically refers to the time spent on screen-based behaviours that can be performed while being sedentary or physically active [1]. In recent years, there has been a significant increase in the accessibility of digital technologies and a potentially worrying trend for increasing use of these devices and its associated sedentary screen time, mainly for the young generations [2]. Increased screen time has been found to be related to a number of negative health and wellbeing indicators in youth populations. Specifically, authors have highlighted the negative health association with adiposity [3,4,5,6,7,8] and cardiorespiratory fitness [4,5], depression and mental health [5,9], diet and quality of life [5], lower academic achievement [5,10], sleep [5,11], and lower levels of physical activity [3,6].

Andersen et al. [12], in a nationally representative sample (n = 2964) of US children aged 4–11 years, highlighted age (older), gender (female), and BMI-for-age ≥95th percentile as characteristics that increase the probability of high screen time. Additionally, there is a shift in gender-related health status after childhood, with females notably decreasing in wellbeing [13]. O’Brien et al. [14] highlighted an inverse relationship between screen time and overall wellbeing, but no relationship between screen time and BMI, in Irish children of social disadvantage (n = 705, aged 7–9 years). Lane et al. [15] reported from a nationally representative sample of 8568 nine year old Irish children that screen time increased the risk of overweight and obesity. Finally, there is a growing recognition of the influence that wellbeing and BMI have on one another, with results indicating that obesity has a strong negative influence on wellbeing [16].

Self-reported screen time is consistently higher in boys than girls [8,17,18,19]. Reported daily screen time appears to increase with age [8,17], and specific screen behaviours also vary with age [19,20]. The home environment plays a significant role in screen time behaviour, with research highlighting that lower socioeconomic home environments provide more opportunities for sedentary behaviour such as media access in bedrooms, but lower access to portable activity equipment and more restrictive rules about physical activity participation [2,21]. It is also important to question how this rapid transformation in digital engagement may affect the health and wellbeing of children [2].

There remains consistent evidence for the relationship between screen time and negative health and wellbeing outcomes, and the tracking of these behaviours from childhood to adulthood [4,5,8,20,22]. Globally, this has led to increased interest across public health and practise, with many countries adopting specific screen time guidance for children in national policy, in most cases embracing a ‘less is better hypothesis’ [20] (p. 2). There is debate regarding the inclusion of quantitative targets as the evidence base is still considered weak [23], while others argue that setting such guidance is low risk and important for public health [24]. In Ireland, the Health Service Executive does not offer a specific recommendation on screen time in children, but currently suggests that limits should be placed on time spent on screens for children aged 6 and older [25]. This guidance, though non-specific, is in line with recent updates from the World Health Organisation (WHO, Geneva, Switzerland). The 2020 WHO guidelines [26], which now include both physical activity and sedentary behaviour, state that children and adolescents are recommended to “limit the amount of time spent being sedentary, particularly the amount of leisure screen time” (p. 1456). It is reported that there was insufficient evidence to set a precise threshold (or ‘cut-off’) for the amount of sedentary or leisure screen time in any age group or ability [26], which suggests there is a need for more research in this area, particularly regarding the impact of quantity and quality of screen time on health and wellbeing.

The most recent analysis from the WHO Childhood Obesity Surveillance Initiative, indicated that on average, 60.2% of children aged 6–9 years old were engaged in screen time <2 h per day, with 14.6% spending 3 h or more engaged in screen time [27]. In Ireland, the most recent CSPPA (Children’s Sport Participation and Physical Activity) study reported that on average, primary school pupils spent five hours per day in sedentary leisure time (including sedentary time and homework), with this rising to 6.9 h a day in post-primary pupils [28]. Findings from this same report, which adopted a 2-h threshold for sedentary screen time within their analysis, found that 63% of primary school aged children were meeting the recommendation of less than two hours of screen time per day, but this dropped to just 42% of post-primary school children. This fall off in the proportions meeting the screen time guidelines as children age is consistent with the previous iteration of this study [29].

Establishing possible causes and outcomes on wellbeing is key within childhood [30], with calls for more research in screen time before limits are established [31]. Longitudinal studies have found that increases in leisure screen time precede lower psychological wellbeing [30,32,33,34]; building on this foundation and understanding the other dimensions of children’s wellbeing will provide clarity, alongside a deeper and broader knowledge that can shape policy and guidelines. Recently published work [2] compared two cohorts of the Growing Up in Ireland study. The longitudinal data provided key insights into the changing digital landscape and its impact on young people in Ireland, with the 2017/18 cohort more active on digital devices and social media compared with the 2007/08 [2], which is reflective of international trends [35]. Findings from the Growing Up in Ireland Study also indicated that spending more than 3 daily hours on TV/digital activities was associated with significant declines in child socioemotional wellbeing, while such effects were stronger in 2017/18 than in 2007/08 [2]. Media engagement was also associated with moderate declines in socioemotional wellbeing, both in 2007/08 and in 2017/18 [2]. Previous research demonstrating the relationship between screen time and wellbeing has indicated small associations, with researchers in these areas citing difficulty in assessing these constructs in children and young people [31,36,37]. As a result, further exploration of the relationship between screen time and wellbeing in Irish children is warranted.

Based on current knowledge, the objective of this study is to determine the effect of screen time and gender on children’s wellbeing in Irish primary school children, with a view to providing evidence to inform and improve the efficacy of future screen-time interventions, including informing future policy development.

## 2. Materials and Methods

### 2.1. The Participants

A convenience sample of children was recruited. Assistance in identifying schools was provided by coaches who were working closely with the schools at the time through their role as games development officers with a large sport’s national governing body (Gaelic Athletic Association, Dublin, Ireland). Principals from all 30 schools consented to take part. Children from third to sixth class across these schools were invited to participate. Participating children and their parents were given plain language statements to confirm they understood the purpose of the research and participation rights (e.g., voluntary participation, right of withdrawal, and confidentiality of the data). Participating children completed assent forms, while parents signed informed consent forms. Ethical approval was granted by the Dublin City University Research Ethics Committee to collect data from February to June 2017 (DCUREC/2017/029). A total of 1053 children from a possible sample of 1104 consented to participate; after participants with missing data were removed (due to a technical error participants’ entire data were missing), the final sample consisted of 897 children, 24% attended schools that are part of a national programme aimed at addressing educational needs in disadvantaged communities.

### 2.2. Procedures and Measures

For all measures, a minimum ratio of 1 researcher to 5 children was employed. All measures were taken during the school day. In order to determine body mass index (BMI), weight was measured to the nearest 0.1 kg using a Salter Professional Analog weighing scales. Height was measured to the nearest millimetre using a portable Leicester stadiometer. The formula used to calculate BMI was kg/m^2^ where kg is a person’s weight in kilograms and m^2^ is their height in metres squared. Adhering to ethical gender protocol when measuring childhood height and weight, two field staff were trained by the principal investigator prior to data collection. Participants had these measurements taken in groups of four at a time, behind a partition, with precautions in place to ensure measurements were taken in a sensitive manner.

The questionnaire, as described below, was completed on tablets (8” display; The Alcatel PIXI 3) via ‘Survey Anyplace’ in the children’s classroom. Children were encouraged to take their time, reflect on their answers, and to be as honest as possible. The first element of the questionnaire, the KIDSCREEN-27 [38], was employed as a measure of wellbeing. This instrument has previously been used to assess wellbeing in studies of primary school children in Ireland [14,39], with those studies yielding satisfactory confirmatory fit indices and internal consistency scores. KIDSCREEN-27 examines five dimensions of health-related quality of life (HRQoL), (i) physical wellbeing, (ii) psychological wellbeing, (iii) parents and autonomy, (iv) peer acceptance, and (v) school and learning. A 5-point Likert scale was used, with responses of “never”, “seldom”, “sometimes”, “often”, and “always” (scoring 1–5) available to participants. The five KIDSCREEN-27 dimensions were shown to have satisfactory internal reliability in this study, with Cronbach α ranging from 0.7 to 0.79.

Leisure screen time was obtained via a self-report questionnaire [40]. Children were asked to think about a normal week, during school term, and to report how long they usually spent engaged in leisure screen time (time spent watching television or videos, playing computer or video games, and playing on a smartphone or tablet) before and after school on each day of the week and on each day of the weekend.

### 2.3. Data Processing

Negatively formulated KIDSCREEN questions were all recoded to have scorings from 1 to 5 with higher values indicating a higher wellbeing. Question scores of the five wellbeing dimensions were summed up. Raw data were converted to Rasch person parameter estimates and then transformed into z-values, and subsequently transformed into T-value scores [41]. With the intention of assessing wellbeing as a multivariate construct, a total wellbeing score was calculated by summing the five wellbeing dimensions together. Higher scores indicated a more positive wellbeing.

Leisure screen time (mean hours per day) was calculated as the daily mean time (hours) spent watching television or videos, playing computer or video games, and playing on a smartphone or tablet. For the purpose of comparison against the range of thresholds employed in other national and European studies [15,27,28], screen time hours were then categorised into a number of groups; <1 h/day, 1–3 h/day, <2 h per day, and 3+ hours per day. While no specific screen time limit is set by the Irish Health Service Executive, two hours as a threshold for categorising screen time was selected for use in statistical analysis for consistency with other national and international studies [27,28] and recent 24-h movement guidelines published internationally (Australia [42] and Canada [43]). BMI was calculated using the equation weight (kg)/height^2^ (m).

### 2.4. Data Analysis

Data were analysed using SPSS version 27 for Mac. Descriptive statistics and frequencies were calculated for leisure screen time (±two-hour threshold), overall wellbeing, five wellbeing dimensions (physical, psychological, parental, peers, and school), and BMI.

Initially, a two-way ANCOVA was conducted to examine the interaction effect of leisure screen time threshold (IV; <2 h vs. >2 h) and gender (IV; girls vs. boys) on overall wellbeing, after controlling for BMI (covariate). Follow-up, one-way MANCOVAs were run to determine the individual effect of the leisure screen time thresholds and gender on the five separate dimensions of wellbeing. The first MANCOVA investigated the effect of leisure screen time thresholds on the five dimensions measures of wellbeing (physical, psychological, parental, peers, and school) while controlling for BMI. The second MANCOVA investigated the effect of gender on the five dimensions measures of wellbeing (physical, psychological, parental, peers, and school) while controlling for BMI. When multivariate effects were significant, univariate analyses were performed to examine which dimensions of wellbeing were significantly different between screen time threshold groups (<2 h vs. 2 h +) and gender (females vs. males). Effect-size measures were presented for the comparison analyses, considering partial η^2^ ≥ 0.01, partial η^2^ ≥ 0.06, and partial η^2^ ≥ 0.14 as small, medium, and large effects, respectively [44].

## 3. Results

### 3.1. Summary Statistics

Participants in this study (n = 897) were 47.7% female, with a mean age of 10.9 ± 1.16 years, and a mean BMI of 18.32 ± 3.77 kg/m^2^ (18.3 ± 3.72 males, 18.35 ± 3.82 for females). Participants accumulated a mean of 1.55 ± 0.88 h/day of leisure screen time; 1.67 ± 0.89 h for males, and 1.41 ± 0.85 h for females. Overall mean (± SD) wellbeing score for the sample was 248.79 ± 31.45, with male and female means being 245.89 ± 31.45 and 252.01 ± 29.78, respectively. Table 1 presents the percentage of participants overall, and by gender, accumulating <1 h, <2 h, 1–3 h, and 3+ h of leisure screen time/day.

### 3.2. Interaction Effect of Leisure Screen Time and Gender on Overall Wellbeing, Controlling for BMI: Two Way-ANCOVA

A two-way ANCOVA was conducted to examine the interaction effect of leisure screen time thresholds (IV; <2 h vs. ≥2 h) and gender (IV; girls vs. boys) on overall wellbeing, after controlling for BMI (covariate).

There was a linear relationship between BMI and overall wellbeing for each group, as assessed by visual inspection of a scatterplot. There was homogeneity of regression slopes as determined by a comparison between the two-way ANCOVA model with and without interaction terms, *F*(3, 889) = 1.124, *p* = 0.339. There was homoscedasticity within groups, as assessed by visual inspection of the studentized residuals plotted against the predicted values for each group. There was homogeneity of variances, as assessed by Levene’s test of homogeneity of variance (*p* = 0.868). There were two univariate outliers in the data, as assessed by studentised residuals. Both outliers were assessed for data entry and measurement errors and deemed genuinely unusual values [45]; neither were removed from the analysis. There were no leverage or influential points, as assessed by leverage values and Cook’s distance, respectively. Studentised residuals were normally distributed, as assessed by the Shapiro–Wilk’s test (*p* > 0.056).

Results of the two-way ANCOVA indicated there was not a statistically significant two-way interaction between leisure screen time thresholds and gender on overall wellbeing, whilst controlling for BMI, *F*(5, 888) = 0.446, *p* = 0.817, Pillai’s = 0.003, partial η^2^ = 0.003 (BMI as a covariate was evaluated at 18.3225). On this basis, the independent variables were analysed individually using two one-way MANCOVA’s.

#### 3.2.1. Leisure Screen Time Threshold Differences in Wellbeing, Controlling for BMI: One-Way MANCOVA

Table 2 shows the breakdown of wellbeing scores (mean/adjusted mean and SD/SE) across screen time category (below or above the 2-h threshold). A one-way MANCOVA was conducted to examine the differences between participants who were below or above the 2-h leisure screen time threshold, on the five dimensions of children’s wellbeing, after controlling for BMI.

There was a linear relationship between physical, psychological, parental, peers, and school wellbeing for each leisure screen time group, as assessed by visual inspection of a scatterplot. There was homogeneity of regression slopes, as assessed by the interaction term between weight and group, *F*(5, 889) = 0.930, *p* = 0.461. The assumption of homogeneity of covariance matrices was violated, as assessed by Box’s M test (*p* < 0.001). Pillai’s Trace criterion rather than Wilks’ Lambda was therefore used, as it is more robust to unequal covariance matrices [46]. Due to the unbalanced sample size design, a lower level of statistical significance (0.01 α level) was set for the one-way MANCOVA. For consistency, Pillai’s Trace criterion and a lower level of statistical significance (α level) were used for both one-way MANCOVAs and all follow-up analysis. There were 19 univariate outliers in the data, as assessed by standardised residuals greater than ±3 standard deviations, and 15 multivariate outliers in the data, as assessed by Mahalanobis distance compared against a chi-square (χ^2^) distribution 20.52 (*p* > 0.001). All outliers were assessed for data entry and measurement errors, and deemed genuinely unusual values; as such, none were removed from the analysis [45]. Residuals were not normally distributed, as assessed by the Shapiro–Wilk’s test (*p* < 0.05), common in unequal groups [45].

The one-way MANCOVA showed that there was a statistically significant difference between leisure screen time groups on the combined dependent variables after controlling for BMI, *F*(5, 890) = 7.453, *p* < 0.0001, Pillai’s = 0.040, partial η^2^ = 0.040. Follow-up univariate one-way ANCOVAs were performed. A Bonferroni adjustment was made such that statistical significance was accepted at *p* < 0.002.

There were statistically significant differences in adjusted means for physical wellbeing (*F*(1, 894) = 27.344, *p* < 0.0001, partial η^2^ = 0.030), parental wellbeing (*F*(1, 894) = 17.805, *p* < 0.0001, partial η^2^ = 0.020), peers wellbeing (*F*(1, 894) = 9.123, *p* = 0.002, partial η^2^ = 0.010), and school wellbeing (*F*(1, 894) = 18.180, *p* < 0.0001, partial η^2^ = 0.020), but not for psychological wellbeing (*F*(1, 894) = 1.799, *p* = 0.180, partial η^2^ = 0.002). Statistically significant one-way ANCOVAs were followed up with pairwise comparisons with the Bonferroni adjustment (see Table 3).

Pairwise comparisons with a Bonferroni-adjusted *p*-value were made for the wellbeing dimensions of physical, parental, peers, and school wellbeing. Adjusted mean and differences based on estimated marginal means are presented (see Table 2). Physical wellbeing was statistically significantly greater within leisure screen time thresholds (*M_adj_* = 53.36, *SE* = 0.33) compared to those over leisure screen time thresholds (*M_adj_* = 48.73, *SE* = 0.61), a mean difference of 3.63, 95% CI [2.267, 4.991], *p* < 0.001. Parental wellbeing was statistically significantly greater within leisure screen time thresholds (*M_adj_* = 51.38, *SE* = 0.38) compared to those over leisure screen time thresholds (*M_adj_* = 48.03, *SE* = 0.70), a mean difference of 3.35, 95% CI [1.793, 4.912], *p* < 0.001. Peer wellbeing was statistically significantly greater within leisure screen time thresholds (*M_adj_* = 54.32, *SE* = 0.38) compared to those over leisure screen time thresholds (*M_adj_* = 51.91, *SE* = 0.70), a mean difference of 2.41, 95% CI [0.845, 3.978], *p* = 0.002. School wellbeing was statistically significantly greater within leisure screen time thresholds (*M_adj_* = 55.67, *SE* = 0.37) compared to those over leisure screen time thresholds (*M_adj_* = 52.34, *SE* = 0.69), a mean difference of 3.317, 95% CI [1.790, 4.844], *p* < 0.001. All other pairwise comparisons were not statistically significant (see Table 3).

#### 3.2.2. Gender, Controlling for BMI Differences in Wellbeing: One-Way MANCOVA

Table 4 shows the breakdown of wellbeing scores (mean/adjusted mean and SD/SE) across gender. A one-way MANCOVA was conducted to examine gender differences on the five dimensions of children’s wellbeing, after controlling for BMI.

There was a linear relationship between physical, psychological, parental, peers, and school wellbeing for girls and boys, as assessed by visual inspection of a scatterplot. There was homogeneity of regression slopes, as assessed by the interaction term between BMI and gender, *F*(5, 889) = 0.974, *p* = 0.433. The assumption of homogeneity of covariance matrices was violated, as assessed by Box’s M test (*p* < 0.001). The adjustments as described in Section 3.2.1 were again applied. Residuals were not normally distributed, as assessed by the Shapiro–Wilk’s test (*p* < 0.05). Equal sample sizes between female and males required interpretation of Normal Q-Q plots; the deviations from the straight line were minimal, indicating normal distribution.

The one-way MANCOVA showed that there was a statistically significant difference between genders on the combined dependent variables after controlling for BMI, *F*(5, 890) = 7.366, *p* < 0.0001, Pillai’s = 0.040, partial η^2^ = 0.040. Follow-up univariate one-way ANCOVAs were performed. A Bonferroni adjustment was made such that statistical significance was accepted at *p* < 0.002.

There were statistically significant differences in adjusted means for peers wellbeing (*F*(1, 894) = 8.722, *p* = 0.002, partial η^2^ = 0.010) and school wellbeing (*F*(1, 894) = 27.103, *p* < 0.0001, partial η^2^ = 0.029), but not for physical wellbeing (*F*(1, 894) = 0.005, *p* = 0.941, partial η^2^ < 0.001), psychological wellbeing (*F*(1, 894) = 2.6, *p* = 0.107, partial η^2^ = 0.003), or parental wellbeing (*F*(1, 894) = 0.602, *p* = 0.438, partial η^2^ = 0.001). Statistically significant one-way ANCOVAs were followed up with pairwise comparisons with the Bonferroni adjustment (see Table 2).

Pairwise comparisons with a Bonferroni-adjusted *p*-value were made for the wellbeing dimensions of school and peer wellbeing. Adjusted mean and differences based on estimated marginal means are presented (see Table 3). School wellbeing was statistically significantly greater in girls (*M_adj_* = 56.70, *SE* = 0.47) compared to boys (*M_adj_* = 53.32, *SE* = 0.45), a mean difference of 3.370, 95% CI [2.1, 4.641], *p* < 0.0001. Peer wellbeing was statistically significantly greater in girls (*M_adj_* = 54.81, *SE* = 0.48) compared to boys (*M_adj_* = 52.84, *SE* = 0.46), a mean difference of 1.972, 95% CI [0.662, 3.283], *p* = 0.002. All other pairwise comparisons were not statistically significant (see Table 3).

## 4. Discussion

This study investigated the effect of leisure screen time and gender on wellbeing in a cohort of primary-school-aged children. While the results of the current study show this Irish sample to compare favourably with Swedish counterparts in the physical wellbeing domain, Irish children recorded lower scores in the remaining domains [47]. With research emanating from Europe reporting that wellbeing decreases with age [47], the fact that these wellbeing levels or Irish children are lower in comparison is concerning, while reinforcing the idea that this trend is happening at scale.

Females scored significantly greater in the social support and peers wellbeing dimension and the school wellbeing dimension. The non-significant interaction effect highlights that gender differences/similarities in wellbeing do not depend on leisure screen time. Females having stronger social relations with peers aligns with previous gender difference research [13,14]. This dimension explores the quality of the interaction between children and their peers as well as their perceived support, with a high score indicating feeling accepted, supported, and included in a peer group [41]. Being accepted and supported by peers is linked to a child’s skill at navigating cooperation, compromise, and competition successfully [48,49]. Gender differences in said skills are well established in the empirical literature, with girls consistently presenting higher levels of support and intimacy, as they consider their peers’ perspectives, empathise, and offer softened communication skills such as agreements and acknowledgement to their peers [48,49]. Moreover, peer support has been shown to be related to higher self-worth [50], lower levels of depressive symptoms and loneliness [51], and higher school satisfaction [52]. In contrast, competitiveness at school is related to poor school wellbeing [53].

The gender difference in school wellbeing is once more consistent with the literature [13,14]. This dimension explores a child’s perception of their cognitive capacity, learning, and concentration, and their feelings about school. In addition, the dimension explores the child’s view of the relationship with their teachers [41]. In educational research, teachers’ support has generally been found to be related to students’ health-related quality of life [54]. Support from teachers is considered of great importance for boys’ school wellbeing, whereas for girls, teacher support has been found to have no influence on their school wellbeing [55]. It is interesting to note that perceived loneliness in school does have an adverse effect on girls’ school wellbeing [55], again highlighting that the quality of the interaction between girls and their peers is key to their development. Meanwhile, it is argued that boys’ tendency to be competitive can lead them to being prone to academic failures, thus needing teacher support to reassure them and maintain their school wellbeing [55,56]. In fact, studies indicate that how boys evaluate their abilities, and themselves as people, does depend on the support and feedback of their teachers [54].

Findings of this study show that Irish children spend an average of 155 min in front of a screen daily for leisure activities. This compares with 91–120 min reported by Healy et al. [57] for typically developing Irish children. O’Brien et al. [14] reported a much higher average screen time of (2.65 h) in eight-year-old Irish children; however, notably this study focussed on children from areas of social disadvantage, with children from lower socioeconomic background known to be of higher risk of negative health implications associated with increased screen time [2]. Results of the current study are relatively consistent with that of Lane et al. [15], a study with a representative sample of 9-year-old Irish children, the notable exception being that a smaller proportion of males in the current study reported to accumulate <1 h screen time daily (16.7% versus 25.5% in Lane et al. study), with a larger proportion accumulating 1–3 h daily (73.4% compared to 65.2% in the Lane et al. study). The reason for this difference is not clear. It may be due to the slightly older age group of the current study (10.86 ± 1.16 years), and as such may support the age-related increase in screen time behaviour reported by Woods et al. [28]. The current study suggests that Irish youth accumulate less screen time than international peers. For example, LeBlanc and colleagues [17] examined cohorts of similar age in several countries and found children accumulated an average of 2.8 h daily, with countries such as Australia (2.8 h), Canada (2.4 h), and the UK (2.8 h) all reporting higher screen time among their respective children. Such differences should be interpreted with caution however, given the variation in screen time measures used. A recent systematic review of the measurement of screen time in young children concluded that whilst studies have generally evolved to reflect children’s contemporary digital landscape, the transparency, description, and appropriateness of some of the screen time measures adopted can require greater clarity, particularly when reporting methodology and findings [58].

Results of the current study also show that boys engage in significantly more screen time than their female counterparts (1.67 h versus 1.41 h) which, again, is consistent with previous findings [15,59]. Boys have repeatedly been shown to accrue more screen time and be less likely to adhere to screen time thresholds than girls [60,61,62]. Again, recent Irish research reports boys from disadvantaged schools accumulating significantly more screen time (3.01 h) than girls (2.27 h) [14]. With screen time purported to be a key determinant of sedentary behaviour [63], these findings warrant attention in the context of children’s health. In the advent of technology advancements and increased accessibility, there is rising concern that increased screen time will have adverse effects on children’s physical health and wellbeing [2,8,14]. 

Previous systematic reviews [5,64] have reported an inverse relationship between total screen time and self-reported wellbeing. The current study findings support that children who accumulated <2 h/day score higher in overall wellbeing than those that exceed the threshold. Often, cross-sectional data, such as that in the current study, would make it difficult to state with absolute certainty whether screen time is leading to poorer wellbeing or in fact whether poorer wellbeing influences children to choose more sedentary pursuits such as screen time [65]. However, the strength of the multivariate analysis utilised in this study does allow for inference causality, and, consistent with other authors, supports the directionality of screen time influencing wellbeing [64,66].

As emerging policies around screen time suggest ‘less is better’ [20], but sufficient evidence is not yet accrued to inform a precise health related threshold (or ‘cut-off’) for leisure screen time [26], the current study adds to the evidence base supporting that those that accrue <2 h leisure screen time daily have significantly higher wellbeing. Specifically, when analysed by domains, results point to significant differences between those that meet and do not meet that 2-h threshold, with those children accumulating less than two hours of leisure screen time per day scoring significantly higher in the physical, social, peer, and parental wellbeing domains than those that do not meet this threshold. This study supports that thresholds have a role to play, given the poorer wellbeing outcomes observed for those children accruing more than two hours/day.

Consistent with the current study, a recent narrative review by [67] also concluded that higher levels of screen time are generally associated with poorer mental health outcomes among children and adolescents, but highlighted that the associations are influenced by screen type, gender, and age. The review indicated that for adolescents and children, there were many cross-sectional studies supporting the negative association between screen time and psychological wellbeing, such as is the case with the current study. The review further identified several longitudinal papers reporting that higher volume of leisure screen time predicted lower psychological wellbeing in children and adolescents. Authors [67] reported that gender differences were apparent in the studies reviewed, dependent on the specific type of screen behaviour, with the negative relationship between social media and wellbeing higher in females, and the negative relationship between video gaming and wellbeing higher in males. It is suggested that the mechanisms underpinning these differences may well reflect differences in screen use between males and females. As the current study did not use the same screen time measures, it is not possible to draw comparisons, but is an important consideration for future study. A recent study with an adolescent 13–15 year olds sample [68] also supports the negative association between screen time and mental health, and furthermore highlights the differential effect of specific screen time behaviours (consistent with [67]) on mental health indicators; pointing again to the importance of measurement transparency and clarity as outlined by [58]. In the Twenge and Farley [68] study, it was shown that screen time behaviours of social media and Internet use were more strongly associated with the mental health indicators measured (self-harm behaviour, depressive symptoms, life satisfaction, and self-esteem) than electronic gaming and TV watching. Furthermore, they found this association stronger in females than males, with heavy female Internet users 166% more likely to have clinically relevant levels of depressive symptoms than low female users, compared to 75% more likely in males. This is also consistent with findings reported in the Mougharbel and Goldfield [67] review. While acknowledging that children of the age of this study’s participants are less likely to spend significant screen time on social media or Internet for leisure time, the use of a screen time measure with sufficient detail to allow for analysis similar to Twenge and Farley [68] would be beneficial in future research (i.e., a breakdown into these same categories of leisure screen time).

It is important to consider screen time and wellbeing throughout childhood and adolescence, with early adolescence highlighted (identified as 10–15 years in that study) as a crucial period for the development of unhealthy behaviours related to psychological wellbeing [69]. Mougharbel and Goldfield [67] acknowledged that in their review most of the studies available were conducted with adolescents, and highlighted that the evidence base among pre-adolescence and young children is only moderate. The current study adds to this evidence base for pre-adolescent youth. Mougharbel and Goldfield [67] also underlined the need for further longitudinal examination of the relationship between screen time and psychological wellbeing in order to better understand the association. The authors recommended that researchers, parents, practitioners, and policy makers should identify and evaluate strategies to reduce screen time in children and adolescents, or strategies to encourage young people to use screens adaptively, in order to promote better mental health in this cohort [67]. In order to do this, however, and to really develop and identify targeted strategies with greatest capacity for impact, longitudinally investigating and reporting patterns of screen time behaviours in childhood up through adolescence, and patterns of associations between such behaviours and psychological health and wellbeing, is critical to understanding the evolution and implications of screen time behaviours in young people.

The strengths of this study include the large representative samples size, the use of multivariate analysis, and the inclusion of BMI as a covariate in analysis. However, limitations should also be highlighted, with the lack of information regarding type of screen behaviour being of note in this regard and other baseline characteristics (e.g., parental education, household income, and race). Research conducted after the current paper’s data collection has indicated that self-reported screen time is inconsistent [69,70]. Further research is required to contribute to the growing evidence base and inform the setting of a precise leisure screen time threshold which can be consistently applied across countries internationally. Moreover, there is an opportunity to determine if leisure screen time predicts wellbeing, wellbeing predicts screen time, or if it is bi-directional. In addition, investigation into why four of the five domains of wellbeing are significantly impacted by leisure screen time but psychological wellbeing is not is also warranted. Further research is also required to better understand the societal factors influencing the differences observed in females’ and males’ wellbeing dimensions. It also seems important to consider how the dynamics between the several domains may change over time from childhood to adulthood.

## 5. Conclusions

Children who self-reported less than 2 h of leisure screen time scored significantly higher on four dimensions of wellbeing (physical, parental, peers, and school, but not psychological) than those that reported 2 h or more. This study supports the growing evidence of the impact that leisure screen time has on health and underlines the differential health profile of children in terms of psychological wellbeing across screen time thresholds. Internationally, defining clear guidelines on hourly leisure screen time limits for children and young people should be an urgent priority, with growing evidence supporting such differential health profiles when a threshold is used. Such guidelines, if applied consistently internationally, would also allow for clearer comparison of patterns and behaviours across countries.

## Figures and Tables

**Table 1:** Percentage of participants across range of screen time categories.

	<1 h	<2 h	1–3 h	3+ h
Overall	22.10%	69.60%	70%	7.90%
Male	16.70%	65%	73.40%	9.90%
Female	28.10%	74.70%	66.30%	5.60%

Note: Screen time categories are not distinct. They are selected based on categories employed in previous studies, and as such, there is some overlap.

**Table 2:** Mean and adjusted mean for the five wellbeing dimensions with and above 2-h screen time threshold. One-way MANCOVA.

	Screen Time	<2 h	2 h +
Physical	*M (SD)*	52.35 (8.80)	48.73 (8.22)
	*M_adj_ (SE)*	52.36 (0.33)	48.73 (0.61)
Psychological	*M (SD)*	38.05 (3.46)	37.66 (4.41)
	*M_adj_ (SE)*	38.05 (0.14)	37.65 (0.26)
Parental	*M (SD)*	51.39 (10.10)	48.02 (9.39)
	*M_adj_ (SE)*	51.38 (0.37)	48.03 (0.70)
School	*M (SD)*	55.66 (9.64)	52.33 (10.02)
	*M_adj_ (SE)*	55.66 (0.37)	52.34 (0.69)
Peers	*M (SD)*	54.32 (9.73)	51.90 (10.81)
	*M_adj_ (SE)*	54.32 (0.38)	51.91 (0.70)

Note. Means (*M*), adjusted means *M_adj_*, standard deviations (*SD*), and standard errors (*SE*) for the five wellbeing dimensions for leisure screen time thresholds.

**Table 3:** Pairwise contrasts for adjusted means for five wellbeing dimensions.

Differences in Adjusted Means (95% CI)		
Wellbeing Dimension	<2 h vs. 2+ h	Girls vs. Boys
Physical	3.63 [2.267, 4.991] *	0.044 [1.113, 1.200]
Psychological	0.396 [0.183, 0.976]	0.398 [0.086, 0.883]
Parental	3.35 [1.793, 4.912] *	0.521 [0.796, 1.837]
School	3.317 [1.790, 4.844] *	3.370 [2.1, 4.641] *
Peers	2.41 [0.845, 3.978] *	1.972 [0.662, 3.283] *

Note. * = statistically significant (*p* < 0.002) based on Bonferroni adjustment; 95% confidence interval (CI) is simultaneous confidence interval based on Bonferroni adjustment.

**Table 4:** Mean and adjusted mean for the five wellbeing dimensions across gender. One-way MANCOVA.

		Girls	Boys
Physical	*M (SD)*	51.56 (8.98)	51.52 (8.64)
	*M_adj_ (SE)*	51.56 (0.43)	51.52 (0.41)
Psychological	*M (SD)*	38.17 (3.13)	37.77 (4.14)
	*M_adj_ (SE)*	38.17 (0.18)	37.77 (0.17)
Parental	*M (SD)*	50.90 (10.12)	50.38 (9.94)
	*M_adj_ (SE)*	50.9 (0.49)	50.38 (0.46)
School	*M (SD)*	56.69 (9.19)	53.31 (10.1)
	*M_adj_ (SE)*	56.69 (0.47)	53.32 (0.45)
Peers	*M (SD)*	54.81 (9.88)	52.84 (10.03)
	*M_adj_ (SE)*	54.81 (0.48)	52.84 (0.46)

Note. Means (*M*), adjusted means *M_adj_*, standard deviations (*SD*), and standard errors (*SE*) for the five wellbeing dimensions.

## Data Availability

Data supporting the results of this study can be accessed by contacting Sarahjane Belton (sarahjane.belton@dcu.ie).
